# Genetic code redundancy and its influence on the encoded polypeptides

**DOI:** 10.5936/csbj.201204006

**Published:** 2012-03-20

**Authors:** Paige S. Spencer, José M. Barral

**Affiliations:** a Department of Biochemistry & Molecular Biology, The University of Texas Medical Branch, 301 University Blvd., Galveston, TX 77555-0620; b Department of Neuroscience & Cell Biology, The University of Texas Medical Branch, 301 University Blvd., Galveston, TX 77555-0620; c Sealy Center for Structural Biology and Molecular Biophysics, The University of Texas Medical Branch, 301 University Blvd., Galveston, TX 77555-0620

## Abstract

The genetic code is said to be redundant in that the same amino acid residue can be encoded by multiple, so-called synonymous, codons. If all properties of synonymous codons were entirely equivalent, one would expect that they would be equally distributed along protein coding sequences. However, many studies over the last three decades have demonstrated that their distribution is not entirely random. It has been postulated that certain codons may be translated by the ribosome faster than others and thus their non-random distribution dictates how fast the ribosome moves along particular segments of the mRNA. The reasons behind such segmental variability in the rates of protein synthesis, and thus polypeptide emergence from the ribosome, have been explored by theoretical and experimental approaches. Predictions of the relative rates at which particular codons are translated and their impact on the nascent chain have not arrived at unequivocal conclusions. This is probably due, at least in part, to variation in the basis for classification of codons as “fast” or “slow”, as well as variability in the number and types of genes and proteins analyzed. Recent methodological advances have allowed nucleotide-resolution studies of ribosome residency times in entire transcriptomes, which confirm the non-uniform movement of ribosomes along mRNAs and shed light on the actual determinants of rate control. Moreover, experiments have begun to emerge that systematically examine the influence of variations in ribosomal movement and the fate of the emerging polypeptide chain.

## Protein synthesis and the redundancy of the genetic code

**Figure 1:**
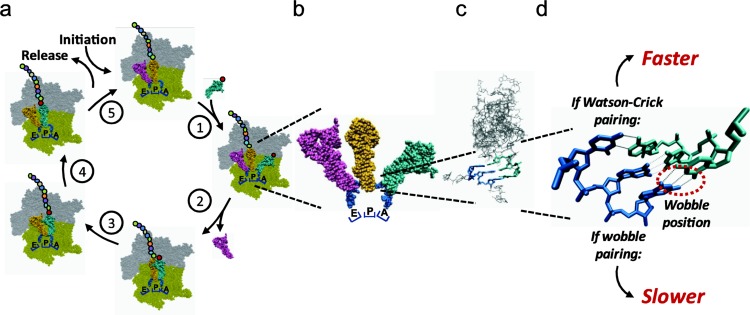
**The nature of the codon:anticodon interaction influences translation elongation**. (**a**) Summary of salient steps during bacterial translation elongation. After initiation, a ternary complex of tRNA (cyan) charged with an amino acid (red dot) and EF-Tu:GTP (not shown) binds to the A site of the 70S complex (gray/green) (1). GTP is then hydrolyzed, which results in incoming tRNA accommodation and release of EF-Tu and deacylated tRNA from the E site (2). The nascent polypeptide (chain of colored dots) is then transferred from the peptidyl tRNA in the P site to the incoming tRNA (3). EF-G binding and subsequent GTP hydrolysis (not shown) results in the critical translocation step, by which the now empty tRNA in the P site is transferred to the E site and the new peptidyl-tRNA is placed in the P site (4). EF-G release now renders the complex competent for a new round of elongation (5) or release and termination, if a stop codon is now encountered in the A site. (**b**) Space filling representation depicting an actual complex of mRNA and tRNAs in the E, P and A sites (PDB file 2Y18, from [76]. (**c**) Stick representation displaying the details of the codon (blue):anticodon (cyan) interaction in the A site shown in b (from [same as above]). (**d**) Enlarged view of actual UGG codon and tRNA^Trp^ anticodon minihelix (PDB file 2Y18 [76]). Wobble position is circled to emphasize that elongation rates will be faster or slower depending on the type of interaction as indicated.

The transfer of genetic information into protein products is termed translation (Figure 1; for detailed reviews on the mechanisms of translation, please see [1–3]). Messenger RNA (mRNA), transcribed from DNA, is translated into protein by a template driven process. The template is composed of a specific combination of 61 trinucleotide codons which encode 20 amino acids. This genetic code is common to most organisms and is referred to as redundant because all amino acids, with the exception of Tryptophan and Methionine, are encoded by more than one codon (termed synonymous codons). Codons are read by adaptor molecules called transfer RNA (tRNA) that bear matching (cognate) trinucleotide sequences, or anticodons. This reading or decoding of the codon occurs by recognition through base pairing, where at least two hydrogen bonds are formed between each of the nucleotide pairs that make up the codon:anticodon minihelix. Only one position of the codon:anticodon minihelix allows pairing that can deviate from standard Watson-Crick (G:C and A:U) interactions. In the third nucleotide of the codon and the first nucleotide of the anticodon, the so-called Wobble position, nonstandard base pairing can occur and results in altered base stacking conformations that are different from that of Watson-Crick pairing yet remain within the conformational constraints of the glycosidic bonds [4]. Interestingly, there are three conserved nucleotides in the bacterial 70S ribosome which maintain decoding fidelity by monitoring the conformation of the bases in the codon:anticodon minihelix [1]. The monitoring of base conformations is much more stringent in the first two nucleotide positions of the minihelix than in the wobble position, allowing for flexibility or wobble in the decoding of this position [1]. For example, nonstandard pairing of G:U and U:G, in which one less hydrogen bond is formed compared to standard G:C and C:G pairing, is allowed only in this position. Furthermore, post-transcriptional deamination of adenosine to inosine in the first anticodon position (INN) expands the decoding capacity from strictly Watson-Crick (A:U) to other allowed “wobble” base pairing (I:U, I:C, I:A) [4]. Adenosine deamination occurs in all eukaryotic ANN anticodons; however, in bacteria, this modification is exclusive to the ACG anticodon of tRNA^Arg^ [5]. There are many other base modifications throughout the tRNA molecule, but these are more variable and will not be considered here. Upon decoding, peptide bond formation is catalyzed in the peptidyl-transferase center of the ribosome and is followed by translocation of the ribosome to the next codon. While diversity exists across evolution in the complexity of the ribosome [1, 6], translation regulation factors [1, 6], and tRNA gene composition [7], the core processes of translation are remarkably conserved and consist of three general steps: initiation, elongation, and termination.

Translation rates are not uniform along an mRNA and vary with the codon composition of the message, since the individual translation rates of codons have been shown to vary by as much as 25-fold [8–10]. The non-uniformity of rates has been proposed to depend on tRNA concentration, the nature of base pairing, and/or mRNA secondary structure [10–12]. The former two will be discussed later in this review. A logical assumption is that a stable mRNA secondary structure may hinder or slow translation by either preventing the ribosome from binding or by acting as a speed bump during ribosomal progression. Indeed, the presence of stable mRNA secondary structures in the ribosomal binding site have been shown to largely affect expression levels as a result of interference with translation initiation [12]. However, the role of mRNA secondary structure in determining polypeptide elongation rates has been disputed [10, 13, 14]. Once the ribosome has initiated translation, it displays powerful helicase activity capable of disrupting very stable mRNA secondary structures (T_m_ = 70°C) [15]. This suggests that mRNA secondary structure plays an insignificant role in the rate of translation elongation, which is the main process addressed in this review. mRNA secondary structure likely plays a much more significant role in translation initiation and termination rates, which will not be discussed here. Additionally, most of the material presented in this review pertains to the bacterial ribosome.

## Polypeptide elongation rate determinants

The process of polypeptide elongation occurs by the sequential addition to the growing polypeptide chain of a single amino acid brought to the ribosome by a molecular complex with three constituents: aminoacyl tRNA (aa-tRNA), elongation factor Tu (EF-Tu), and GTP (a so-called ternary complex) bearing the correct (cognate) anticodon for the mRNA codon in the ribosomal A site (Figure 1). There are three general steps to the elongation cycle: tRNA selection, peptidyl transfer, and translocation. tRNA selection, or decoding, consists of an initial binding of the ternary complex to the ribosome followed by codon recognition. Then, the GTPase activity of EF-Tu is activated, which subsequently causes GTP hydrolysis, EF-Tu dissociation, and accommodation [16]. Accommodation is the movement of the amino acid portion of the aa-tRNA in the A site closer to the peptidyl tRNA in the P site for peptidyl transfer to occur [1]. Following peptidyl transfer, binding of elongation factor G (EF-G) and GTP hydrolysis catalyze the translocation of the ribosome one codon forward, so that the tRNAs now reside in the E and P sites, respectively [1]. The elongation cycle continues as the codon in the newly vacant ribosomal A site awaits the next tRNA arrival. Interestingly, the ribosomal A site is likely seldom vacant and is instead sampled by cognate, near-cognate, and non-cognate tRNAs [17]. The terms, near-cognate and non-cognate, have conventionally been assigned to tRNAs which have single or multiple base mismatches with a given codon, respectively. However, Plant *et al* have challenged that a functional definition, namely the ability to form a minihelix with the codon in the ribosomal A site, better distinguishes a near- from a non-cognate [18]. It is important to note, that as peptidyl transfer and translocation occur much faster, tRNA selection appears to be the rate limiting step of ribosomal progression along the mRNA during polypeptide elongation [10, 19, 20]. Independently, two groups have observed large rate differences in the steps of polypeptide elongation by performing high resolution kinetic studies of the bacterial ribosome *in vitro*. They have determined that the rate of ternary complex GTPase activation in response to codon recognition is the rate limiting step of peptidyl transfer. They found that GTP hydrolysis of the cognate ternary complex occurs 650-fold [16] or approximately 116-fold [21] faster than the near-cognate one (base mismatch in 1^st^ codon position in these studies). The other measurable rates were similar between cognate and near-cognate tRNAs, with the exception of a faster dissociation of the near-cognate during codon recognition [16].

Modeling of this kinetic data agrees with a competition for the A site whereby the binding and rejection of a number of near-cognate tRNAs, prior to the binding and accommodation of the cognate tRNA, delays the rate of translation [17, 22].The faster rate of cognate anticodon recognition combined with the rapid rejection of the near-cognate anticodon emphasize the role of tRNA selection in determining the rate of polypeptide elongation.

Since the binding of the aa-tRNA-containing ternary complex to the ribosome is essentially a binding reaction, concentration of the cognate tRNA for a particular codon should influence the rate at which the ribosome translates that codon. This has indeed been shown by examining the correlation between codon translation rates and cognate tRNA concentrations [10]. Increasing the concentration of tRNA^Trp^ four-fold by overexpression results in a three-fold increase in translation rate of the corresponding codon, UGG [8] (tryptophan is one of only two amino acids which are encoded by a single codon). Most codons can be read by more than one isoacceptor tRNA due to Wobble pairing in the third position of the codon and first position of the anticodon [4]. Conversely, a single tRNA anticodon can decode various synonymous codons, and these can vary in translation rates. For example, the only two codons encoding glutamate, GAA and GAG, are decoded by a single aa-tRNA species at differing rates of 21.6 and 6.4 codons/second, respectively [9] (Figure 1). Similar to GAA and GAG, other *in vivo* measured translation rates of synonymous codons read by identical aa-tRNAs show that those with Watson-Crick pairing in the wobble position are translated faster than those with wobble pairing in every instance [8, 9]. When more than one codon is translated by a single tRNA, the only difference is the nature of the base pairing and base stacking between the third codon position and the first anticodon position. The different rates observed clearly demonstrate that base pairing in the wobble position, in addition to tRNA concentration, determines codon translation rate. Recent ribosomal profiling has solidly corroborated this effect on *in vivo* rates in *C. elegans* and HeLa cells by showing genome wide that ribosomes occupy Wobble read codons for 50% longer than Watson-Crick read codons [14]. Furthermore, out of all NNC and NNU codons, the former are translated faster in *C. elegans* and HeLa cells. This result agrees well with what has been reported previously in *E. coli* [8]. As all NNC/NNU codon pairs are synonymous and can be decoded, in eukaryotes, either by Watson-Crick (G:C), near-Watson-Crick (I:C) or Wobble pairing (G:U or I:U) anticodons (depending on the tRNA gene content of the organism), comparisons of ribosomal occupancy can be derived for certain pairs. Where this was possible, the difference in ribosomal occupancy was greater between Watson-Crick and Wobble than near-Watson-Crick and Wobble [14], implying that rate of codon recognition can be ranked as follows: Watson-Crick > near-Watson-Crick > Wobble.

What might be the advantages that organisms derive from being capable of modulating their translation elongation rates? In addition to enhancing the ability of individual segments of a polypeptide to fold (or avoid misfolding) during translation (please see below), *global* regulation of these rates might be greatly beneficial to cells whose growth is generally regulated by protein synthesis rates according to the “growth optimization model”[23]. It is well known that the process of translation is not absolutely accurate [24]. Yet, various mutations in the bacterial translational apparatus can result in so-called hyperaccurate protein synthesis, where significantly fewer mistakes are made during translation [24]. However, these mutations result in considerably slower rates of polypeptide elongation. In other words, in these mutants, accuracy is achieved at the expense of speed. Thus, it can be concluded that wild type polypeptide elongation rates are a compromise between accuracy and velocity. In circumstances where nutrient availability is limited (and growth is restricted), the cell might need to decrease the production of proteins, yet ensure that those that are synthesized are relatively error free. In opposite circumstances, cells might take advantage of ample nutrients and not be gravely affected by amino acid misincorporation, as errors would be diluted as cells grow and divide.

## Codon bias does not necessarily determine polypeptide elongation rate

**Figure 2:**
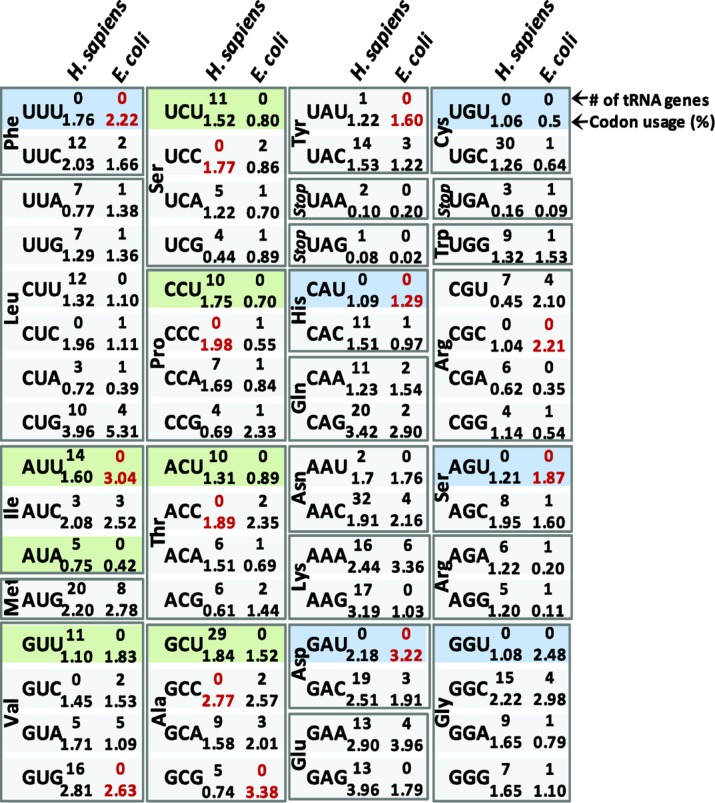
**Differences in tRNA gene content across organisms**. Codons boxed in blue denote tRNA genes often absent in bacteria *and* eukaryotes, while codons boxed in green denote genes mostly absent *only* in bacteria. Actual tRNA gene numbers and codon usage frequencies for humans and *E. coli* are provided as indicated. Numbers in red color denote most frequent codons for which there is no cognate tRNA gene in each organism. Data were were obtained from [7].

As discussed in the above section, it is likely that polypeptide elongation rates depend both on the nature of the anticodon-codon interaction as well as actual aa-tRNA concentrations. The concentrations of tRNA molecules have been experimentally determined for several organisms and cell types, although these measurements do not distinguish between charged and un-charged tRNAs. Regardless, the concentration of particular sets of tRNAs has been shown to correlate relatively well with corresponding tRNA gene numbers. For example, in *E. coli*, the r-values (numerical value describing the linear dependence of datasets such that r = 1.0 indicates a perfect, positive linear relationship) have been reported to vary between 0.74 and 0.9 while in *B. subtillis* r = 0.86 [25, 26]. In the eukaryote *S. cerevisiae*, the correlations reveal a similar dependency: r = 0.91 [27]. Additionally, it is known that there exists some variation in expression of tRNA as a function of growth conditions in both bacteria [28] and unicellular eukaryotes [29]. Regardless of these caveats, tRNA gene number has been largely accepted as a means to estimate relative aa-tRNA concentrations in multiple organisms. It is important to note that correlations have indeed been found between tRNA gene number and the nonrandom use of synonymous codons in highly expressed genes in several unicellular organisms. This has led to the hypothesis that in organisms whose growth rates are largely dependent on the overall rate of protein production, the translation process has been accelerated, and thus optimized, by evolving codon usage in highly expressed genes to match the most abundant tRNAs [11]. In other words, evolving highly expressed genes to largely contain codons read by abundant tRNA would increase the rate of essential protein production and thus increase growth rates in these organisms. These codons were designated as “optimal codons” since they appeared to be favored over their synonymous counterparts in highly expressed genes. Conversely, codons rarely found in highly expressed genes were termed “non-optimal codons” because they were correlated with low abundance tRNAs, although to a lesser extent. Genes with low expression in these organisms, such as those encoding regulatory proteins, were found to be encoded by less biased usage of optimal and non-optimal codons. These results have led to the generalized assumption that frequently used codons are translated fast, and infrequently used codons are translated slowly across organisms, even though the inverse has been shown to occur for some codons [8]. This is perhaps due to the fact that the correlation between codon usage frequency and tRNA availability is clearly not absolute (Figure 2, tabulated from the Genomic tRNA database http://gtrnadb.ucsc.edu/ [7]). For example, highest codon usage frequency and highest tRNA gene number agree only in 11 codons in human and 6 codons in *E. coli*. Furthermore, in most organisms, there are examples in which the most frequently used codon for a particular amino acid across the genome has zero Watson-Crick-decoding tRNA genes and thus must rely on a tRNA that decodes via non-Watson-Crick interactions, which, as mentioned above, is generally slower. For example, in *E. coli* and human, there are 9 and 4 cases, respectively, where the most frequently used codon for a particular amino acid has zero Watson-Crick-decoding tRNA genes (Figure 2). Furthermore, there are several instances where there are vastly more tRNA genes for a particular codon, but the frequency with which that codon is used is only slightly higher (for example, the codons for Asn in humans, Figure 2). It is important to note here that there are different ways in which a codon can be designated as “frequent” or “rare”. The original studies derived codon frequencies from *only highly expressed genes*, whereas modern databases (such as the one utilized to generate Figure 2) tabulate frequencies based on the total appearance of codons *across entire genomes*. There would undoubtedly be more agreement between high tRNA abundance and high usage frequency for *E. coli* if the codon usage data were restricted to highly expressed genes instead of considering all sequenced *E. coli* genes.

The correlation between tRNA abundance and codon usage is maintained for the previously discussed glutamate codons of *E. coli*, as GAA is more frequently used, has more cognate tRNA genes, and is translated faster than its synonymous glutamate encoding counterpart [7, 9]. However, in the same study, the *in vivo* translation speeds of one frequent codon, CCG (Pro), and one rare codon, CGA (Arg), were translated at very similarly slow rates. This is likely due to the low availability of tRNAs to decode these codons (there are 1 and 0 cognate tRNA genes corresponding to these codons, respectively; Figure 2).

These findings and others of the time [11, 30, 31] cultivated an increased emphasis on biased codon usage frequencies in translation speed and evolution studies. In addition to the various datasets that can be utilized to measure codon frequencies, there are multiple formulas by which measures of codon frequency can be calculated, which have led to reports of significantly different usage frequency values [32] and thus variable correlations between “usage frequency” and “speed” [14]. Absolute codon frequency is the number of times a given codon is present in a given gene, set of genes, or an entire genome [33]. The Genomic tRNA database (http://gtrnadb.ucsc.edu/) displays a value for absolute codon usage frequency as a percent of the occurrence of a particular codon throughout all coding sequences available for the organism listed, and does not take into account whether or not that codon is part of a synonymous codon block [7, 34]. An important caveat of this method is that individual amino acids are not equally present in the coding sequences and may introduce an amino acid-related bias in the observed codon usage frequency patterns. In order to represent codon usage bias independently of amino acid bias, relative frequencies can be calculated. Relative codon frequency is the ratio that results from dividing the absolute codon frequency of a particular codon by the sum of the absolute codon frequencies of all codons in a synonymous block [32]. Another codon usage metric, Relative Synonymous Codon Usage (RSCU) [35], takes the calculation one step further by normalizing equal codon usage frequencies within a synonymous block to 1.0 (by multiplying the relative codon frequency by the number of synonymous codons in that block). As stated above, highly expressed genes in bacteria and unicellular eukaryotes tend to be encoded by frequent codons. However, there is no evidence for such bias in the highly expressed genes of vertebrates [11, 14]. Interestingly, in *C. elegans*, genes with high expression were found to be enriched for codons that the authors demonstrate to be translated faster by ribosomal occupancy times [14]. Therefore, the adequacy of codon bias for relative translation rate predictions is limited to highly expressed genes in some unicellular and simple multicellular organisms.

## Polypeptide elongation rates and protein folding

To become biologically active, the great majority of proteins must fold into precise three-dimensional conformations. Invaluable insights regarding how protein chains acquire their so-called native states have come from *in vitro* refolding experiments [36] and computational biology approaches [37]. These studies have demonstrated that the amino acid sequence of a protein encodes in its entirety the necessary information to attain its native state. *De novo* protein folding in the cell differs from *in vitro* refolding in various fundamental aspects, which have just begun to be understood [38, 39]. *In vivo*, proteins emerge gradually from the ribosome as they are being synthesized. Thus, the full-length protein sequence is not available for folding all at once, as it is during *in vitro* refolding. Furthermore, the vectorial nature of ribosomal protein synthesis imparts additional constraints on the folding process. The N-terminus of the protein is always exposed to solvent before its more C-terminal elements, and the rate of appearance of the nascent chain is generally significantly slower (seconds to minutes) than observed rates of *in vitro* refolding (nanoseconds to seconds). Furthermore, in contrast to the optimal conditions prepared for refolding experiments, protein folding in the cell occurs under significant macromolecular crowding and at fixed temperature and ionic strength [40]. In order to allow efficient folding under these conditions, the cell has evolved proteins that assist during *de novo* folding. These proteins, known as “molecular chaperones”, bind reversibly to emerging polypeptides and maintain them in an unfolded ( or partially folded) state until sufficient sequence has been synthesized to form a native domain [41, 42].

The ability to synthesize proteins recombinantly has shown that bacterial systems are often incapable of producing native proteins from human or other eukaryotic origins [43, 44]. The poor capacity of the bacterial cytosol to support efficient folding of certain model proteins has been exploited to investigate the mechanisms and molecules involved in these processes. It is possible that this inability may be due to the presence of incompatible bacterial chaperones [45, 46] or the absence of specialized eukaryotic chaperones [47, 48]. In addition to their distinct chaperone complements, a major difference between the protein biosynthetic machineries of bacteria and eukaryotes that has remained largely unexplored is the rate at which proteins are synthesized. In *E. coli*, polypeptide elongation rates vary from ∼12 amino acids *per* second (aa/s) during slow growth to ∼20 aa/s during fast growth [49]. In contrast, elongation rates in eukaryotes are thought to be fairly constant and considerably slower (∼5 aa/s) [50]. Thus, the folding pathways of nascent polypeptide chains in eukaryotes evolved in the context of synthesis rates slower than those of bacteria. Since translation is spatially and temporally coupled to protein folding, synthesis of certain eukaryotic proteins by bacterial ribosomes at abnormally fast speeds may be incompatible with their folding regimes.

Indeed, it has long been hypothesized that variations in mRNA translation rates could have significant impact on the folding of encoded polypeptides [51, 52] and sequence-based manipulation constitutes a promising strategy to improve the folding of recombinant proteins in heterologous systems [53, 54]. The effect of globally altering translation speeds has been demonstrated by heterologous expression in an *E. coli* strain that has been mutated to produce slow-translating ribosomes [55]. In this study, slow translation resulted in higher folding efficiency of the recombinant proteins compared to those that were translated by faster wild type ribosomes [55]. The effects of regional variations in translation rates on protein folding are generally addressed in two types of approaches: (1) computer-based searches for correlations between codon composition of mRNAs and structural features of the encoded polypeptides; and (2) biochemical investigations of the effects of silent substitutions on the activities of specific proteins (Table 1). These studies have found conflicting results on whether or not certain types of codons encode amino acid residues present in particular structures of the native protein, such as domain boundaries, regions of random coil, or certain secondary structural elements, *etc*. (Table 1). Similarly, there has been disagreement in the literature regarding the effect of “fast” or “slow” codons at certain positions on the solubility and activity of particular proteins (Table 1). These discrepancies are partially due to the fact that most of these studies base translation rate predictions on measures directly related to the above concept of biased codon usage (such as the Codon Adaptation Index [56] and %MinMax [57]), which as stated above, may not accurately reflect polypeptide elongation rates.

**Table 1:** Overview of studies linking mRNA codon composition with protein folding

Year	Protein/Dataset	Methodology	Findings and Remarks	Ref.
1968	Human sickle cell hemoglobin	Theoretical	Proposed “the structure-rate hypothesis and the toll bridge analogy” to explain how a single codon changes along the hemoglobin S molecule could result in misfolding.	51
1987	Feline pyruvate kinase	Theoretical	Correlated the occurrence of rare codons along the pyruvate kinase mRNA with its domain structure. Suggested controlled differential rates of translational elongation as a general mechanism for protein folding *in vivo*.	52
1989	Cytochromes; globins	Theoretical	Observed clusters of rare codons in the boundaries of segments encoding linkers connecting similar secondary structural elements. Suggested that the concentration of tRNA molecules allows sequential domain folding encoded in the mRNA	58
1994	Yeast TRP3	Experimental	Replacement of a segment of ten rare codons in a region predicted to lie between two folding units resulted in decreased specific activity. Removal of SSA (Hsp70) chaperones resulted in a further decrease in activity, supporting the notion of misfolding.	59
1996	37 *E. coli* proteins	Theoretical	Correlated codon frequency with protein domains and found that slow codons clustered around domain boundaries of multi-domain proteins. Utilized a combination of codon frequencies and codon adaptation index to predict translation rates.	60
1996	54 *E. coli* proteins	Theoretical	General trends found for helices to be encoded by codons predicted to be translated fast, and beta strands by codons predicted to be translated slowly. Utilized a combination of codon frequencies and codon adaptation index to predict translation rates.	61
1996	719 proteins from bacteria and eukaryotes	Theoretical	No correlations found between codons predicted to be translated slowly and domain boundaries. Utilized codon adaptation index to predict translation rates.	62
1996	109 mammalian sequences	Theoretical	Found that certain codons have a significantly different propensity for being located at the boundaries of secondary structural elements than the amino acids they encode.	63
1997	Human interferon	Experimental	Replacement of 11 rare Arg codons (AGG, AGA) with a frequent one (CGU) resulted in decreased specific activity upon recombinant production in *E. coli*. Supports idea that increased translation speed increases eukaryotic protein misfolding in *E. coli*.	64
1998	Yeast Ure2p	Experimental	Replacement of two rare Arg (AGA) codons by a more frequent one (CGU) resulted in a significant increment in the yield of biologically active protein upon production in *E. coli*. Does not support the idea that slower translation rates decrease misfolding of eukaryotic proteins in *E. coli*.	65
1999	Bacterial chloramphenicol acetyltransferase	Experimental	Replacement of a segment of 16 rare codons for frequent ones resulted in a 20% decrease in specific activity upon production in *E. coli*. Supports idea that increased translation speed increases protein misfolding.	66
2000	164 proteins from bacteria, yeast and humans	Theoretical	No species-invariant correlation between codon usage and secondary structural elements found, but significant differences for preferred codons found between helices and strands. Utilized synonymous codon usage as predictor of translation rates.	67
2002	cDNas from 21 bacterial species	Theoretical	The location of segments predicted to be translated slowest was mapped and found to be at codon ∼155, proposed to correspond to the emergence of a “typical protein fold”. Translation rate predictions were based on codon frequency.	68
2003	200 proteins from SCOP dataset	Theoretical	Certain codons for Ile and Arg were found to be significantly enriched in folds composed of particular kinds of elements (*e.g*., all alpha proteins). No correlations with predicted elongation rates were attempted.	69
2007	Human P-glycoprotein (MDR1)	Experimental	A silent single nucleotide polymorphism proposed to affect polypeptide elongation rates was found to result in a P-glycoprotein conformation with altered substrate characteristics.	70
2007	HIV *gag* p17	Experimental	A silent substitution in the gag p17 protein in virions incapable of seroconverting human hosts was found to interfere with viral assembly in cell culture models.	71
2009	*E. coli* SufI	Experimental	Correlated putative folding intermediates with regions along the mRNA predicted to be translated slowly. Translation rate predictions were based on a combination of codon frequency and tRNA concentrations.	72
2009	3636 proteins from *E. coli*, yeast, fly and mouse	Theoretical	“Translationally optimal codons” were found to associate with buried residues and with sites where mutations result in large changes in free energy. Translation efficiency was inferred from codon usage bias data.	73
2010	4406 proteins from bacteria and eukaryotes	Theoretical	No evidence found that domain boundaries are enriched in slow codons. However, translation rates predicted to decrease at the transitions into secondary structural elements. Found relative codon usage to be less informative than tRNA concentration for predicting translation rates	74
2010	Mammalian beta and gamma actins	Experimental	Differential arginylation of actin isoforms proposed to occur as a result of sequence-encoded differences in translation rates at the start of the mRNAs, which leads to differential degradation. Translation rate predictions were based on codon frequencies; translation rates were not experimentally determined.	75

How can subtle differences in polypeptide elongation rates impact the folding of the polypeptide emerging from the ribosome? Although 2-3 fold differences in the rates of ordinary reactions might not be generally considered significant from a chemical kinetics point of view, a 2-3 fold difference in the rate of synthesis of a protein may have profound biological consequences. For example, a subtle increase in the concentration of a partially folded, aggregation-prone polypeptide intermediate during translation may exceed the critical concentration of the intermediate and lead to its nucleation-dependent aggregation, thus forming intracellular aggregates. In essence, the finding that variations in translation rates impact protein folding [55] support the notion that not all proteins fold globally, but rather follow particular pathways throughout the available structural space, influenced by the speed at which they emerge vectorially from the ribosome. This idea may find applications in a variety of fields and settings, including improvements in the production of recalcitrant proteins for vaccine development, recombinant pharmaceuticals and structure-determination studies.

Knowledge of the determining factors of polypeptide elongation rates reviewed here should lead to more prudent speed designations for codons and thus more accurate predictions of variations in translation rates along mRNA. This information will help us to understand how this hidden layer of information encoded in mRNA influences the resulting protein structure formation.

## Abbreviations used

A, adenine; A site, aminoacyl tRNA site; aa-tRNA, aminoacyl tRNA; C, cytosine; E site, exit site; EF-G, elongation factor G; EF-Tu, elongation factor Tu; G, guanine; N, nucleoside; P site, peptidyl tRNA site; RSCU, relative synonymous codon usage; T_m_, melting temperature; U, uracil.
